# The Effects of Rhizosphere Inoculation with *Pseudomonas mandelii* on Formation of Apoplast Barriers, HvPIP2 Aquaporins and Hydraulic Conductance of Barley

**DOI:** 10.3390/microorganisms10050935

**Published:** 2022-04-29

**Authors:** Tatiana Arkhipova, Guzel Sharipova, Guzel Akhiyarova, Ludmila Kuzmina, Ilshat Galin, Elena Martynenko, Oksana Seldimirova, Tatyana Nuzhnaya, Arina Feoktistova, Maxim Timergalin, Guzel Kudoyarova

**Affiliations:** 1 Ufa Institute of Biology, Ufa Federal Research Centre, Russian Academy of Sciences, Prospekt Oktyabrya, 69, 450054 Ufa, Russia; tnarkhipova@mail.ru (T.A.); g.v.sharipova@mail.ru (G.S.); akhiyarova@rambler.ru (G.A.); ljkuz@anrb.ru (L.K.); ilshat.rafkatovitch@gmail.com (I.G.); evmart08@mail.ru (E.M.); o_seldimirova@mail.ru (O.S.); tanyawww89@mail.ru (T.N.); feoktistova.arisha@yandex.ru (A.F.); timermax@mail.ru (M.T.); 2 Institute of Biochemistry and Genetics, Ufa Federal Research Center, Russian Academy of Sciences, 450054 Ufa, Russia

**Keywords:** *Pseudomonas mandelii*, *Hordeum vulgare*, apoplast barriers, aquaporins, transpiration, hydraulic conductivity, auxin, indoleacetic acid (IAA), abscisic acid (ABA)

## Abstract

*Pseudomonas mandelii* strain IB-Ki14 has recently been shown to strengthen the apoplastic barriers of salt-stressed plants, which prevents the entry of toxic sodium. It was of interest to find out whether the same effect manifests itself in the absence of salinity and how this affects the hydraulic conductivity of barley plants. Berberine staining confirmed that the bacterial treatment enhanced the deposition of lignin and suberin and formation of Casparian bands in the roots of barley plants. The calculation of hydraulic conductance by relating transpiration to leaf water potential showed that it did not decrease in bacteria-treated plants. We hypothesized that reduced apoplastic conductivity could be compensated by the higher conductivity of the water pathway across the membranes. This assumption was confirmed by the results of the immunolocalization of HvPIP2;5 aquaporins with specific antibodies, showing their increased abundance around the areas of the endodermis and exodermis of bacteria-treated plants. The immunolocalization with antibodies against auxins and abscisic acid revealed elevated levels of these hormones in the roots of plants treated with bacteria. This root accumulation of hormones is likely to be associated with the ability of *Pseudomonas mandelii* IB-Ki14 to synthesize these hormones. The involvement of abscisic acid in the control of aquaporin abundance and auxins—in the regulation of and formation of apoplast barriers—is discussed.

## 1. Introduction

Many rhizospheric microorganisms are capable of stimulating plant growth and increasing their yield, which has led to their being called plant-growth-promoting rhizobacteria (PGPR) [1]. This property opens up the possibility of using PGPR to enhance crop production, attracting the attention of many researchers to studying the mechanisms of their growth-stimulating effects. At the same time, stimulation of plant growth increases leaf area, which can lead to increased evaporation of water from their surface. Indeed, in a number of experiments, the rhizospheric bacteria increased with the rate of plant transpiration [2,3]. Nevertheless, the leaf water content did not change, suggesting the bacteria also increased water delivery from the roots. Since adequate plant hydration is a necessary condition for cell elongation, it is important to understand how the water balance of plants is maintained in plants treated with PGPR. However, the effect of PGPR on plant–water relations has attracted limited attention.

Water flows through the plant depend on the hydraulic conductivity of the apoplast and cell membranes [4]. We recently demonstrated that *Pseudomonas mandelii* accelerated and enhanced the deposition of lignin and suberin and the formation of Casparian bands in the roots of salt-stressed wheat plants [5], which is known to promote salt tolerance in plants by limiting the penetration of toxic ions through the apoplast [6]. Since the formation of apoplastic barriers reduces hydraulic conductivity [7], it was important to check whether PGPR influence the formation of Casparian bands in the absence of salinity and how this affects the hydraulic conductivity of the roots.

When apoplastic hydraulic conductivity is decreased, water flows through membrane water channels, aquaporins, become more important [8]. PGPR alter the activity and expression of plant aquaporins [9]. *Azospirillum brasilense* up-regulated the expression of *HvPIP2;1*-encoding aquaporins of barley seedlings localized in plasmalemma [10], while *Bacillus megaterium* increased the hydraulic conductance by up-regulating the *ZmPIP1;1* and *ZmPIP1;5* expression in maize [11]. While *Pseudomonas mendocina* down-regulated the expression of PIP2 in droughted lettuce plants, it up-regulated it in well-watered plants [12]. These contrasting results indicate that further study of the effects of bacteria on plant aquaporins is needed. Thus, the goal of this work was to study the effects of *Pseudomonas mandelii* strain IB-Ki14 on the formation of apoplastic barriers, the abundance of HvPIP2 aquaporins and the expression of genes encoding them in barley plants to reveal the importance of these effects in regulating plant–water relations.

The species *P. mandelii* is currently insufficiently studied for its potential ability to exhibit PGPR properties. Representatives of the species *P. mandelii* were isolated from natural mineral waters [13,14]. Many strains of *P. mandelii* were isolated from the non-flooded soils of rice fields, comprising almost half (49%) of the 117 isolates of fluorescent pseudomonads, represented by 20 species [15]. One of the strains of *P. mandelii* was isolated from the rhizosphere of *Lolium perenne* [16]. Barley was chosen as a model species due to the availability of antibodies to the N-region of *HvPIP2;1* [17], *HvPIP2;2* [18], *HvPIP2;3* [19] and *HvPIP2;5* [20] and their important role in the regulation of the hydraulic conductivity of the water pathway through membranes [21].

## 2. Materials and Methods

### 2.1. Plant Material, Bacterial Strain and Cultural Media

Barley plants (*Hordeum vulgare* L.) of the prairie variety were used for the study. Barley seeds were sterilized in 2% sodium hypochlorite solution for 10 min. Then, the seeds were repeatedly washed with distilled water, placed in a beaker with water and intensively aerated for 2 h. Seeds were laid out on rafts covered with moist filter paper and kept for 2 days in the dark at room temperature. Plants were grown in 500-mL pots. To enable drainage, gravel was added to the bottom of the pots and glass tubes were installed to ensure gas exchange. The pots were filled with 0.55 kg of sand. Prior to experiments, sand was sterilized by calcinations to exclude the presence of undesirable bacteria. Before planting, the sand was soaked with a 50% Hoagland–Arnon solution to 80% of the total moisture capacity. Ten two-day-old seedlings (with roots about 1 cm long and shoots about 2–3 mm) were placed in each pot and 1 mL of bacterial suspension (10^8^ CFU mL^−1^) was applied to the surface around each seedling, following cultivation of the bacteria as described below. Every other day, the plants were watered with 1 mL of Hoagland–Arnon solution per pot to maintain nutrient status. Substrate water content was kept at 80% by replacing losses (determined by weighing the pots) daily. Plants grown in sand without the introduced bacteria were used as a control. Plants were grown at 14 h photoperiod, illumination of 400 µmol m^−2^·s^−1^ PAR and 25/20 °C (day/night).

Gram-negative bacteria *Pseudomonas mandelii* IB-Ki14 capable of producing auxins were used for inoculation rhizosphere (All-Russian collection of microorganisms, B-3250). Bacteria were cultivated in Erlenmeyer flasks with King’s B medium (2% peptone, 1% glycerol, 0.15% K_2_HPO_4_, 0.15% MgSO_4_·7H_2_O) on a shaker Innova 40R (New Brunswick, NJ, USA) (160 rpm) for 48 h at 28 °C.

### 2.2. Determining the Phosphate-Mobilizing Activity, Antagonistic Activity of the Strain to Microscopic Fungi, Chitinase and Cellulase Activity, Halophilicity of Bacteria

The ability of *Pseudomonas mandelii* strain IB-Ki14 to solubilize orthophosphates of Ca, Al and Fe, as well as salts of inositol phosphoric acid, was measured as described [22]. Antagonistic activity of the strain towards fungal species causing root rot of wheat (*B. sorokiniana*, *F. culmoruim*, *F. oxysporm*, *F. solani*) was tested. Chitinase and cellulase activity was studied on the medium, g/L: NaCl—5, MgSO_4_—2, NH_4_H_2_PO_4_—1, K_2_HPO_4_—1, peptone—3, yeast—3, substrate—5 (carboxymethylcellulose salt or colloidal chitin). Cultures were inoculated by injection on the medium surface. The zone of clearing around the colonies was measured after 7 days for chitinase or antifungal activity, and on day 14 for measuring phosphate mobilizing activity. Evaluation of enzymatic and phosphate-mobilizing activity was calculated as the ratio of the size of the halo zone and colonies [23]. The halophilicity of bacteria was studied in liquid media with NaCl concentration varying from 1 to 10%.

### 2.3. ABA and IAA Assay in Cultural Media

Culture media were immunoassayed on the second day of cultivating bacteria. ABA and IAA were partitioned from culture media of bacteria with diethyl ether as described [24]. In short, one ml of bacterial culture media was diluted with distilled water and acidified with HCl to pH 2.5 to extract ABA and IAA with diethyl ether. Then, hormones were partitioned from diethyl ether into NaHCO_3_ solution and reextracted with diethyl ether from the acidified aqueous phase. ABA and IAA analysis was carried out with enzyme-linked immunosorbent assay using specific antibodies against either ABA or IAA as described [20,25]. The reliability of the method was enabled by specificity of antibodies to hormones and the use of an extraction method that enables efficient recovery of hormones while reducing the amount of impurities by decreasing the volume of extractants at each stage of solvent partitioning. The efficiency of purifying hormones prior to immunoassay was confirmed by the study of their chromatographic distribution, which showed that peaks of immunoreactivity coincided only with the position of the standards of ABA and IAA.

### 2.4. Plant–Water Relations: Transpiration Rate, Water Potential, Hydraulic Conductivity

Pots were covered with parafilm 7 days after bacterial treatment to prevent water evaporating from the surface when measuring transpiration by the weight loss of pots. Leaf (the middle of the first leaf) and soil water potential were measured with L-51 and PST-55 sample chambers, respectively (PSYPRO, “Wescor”, Logan, UT, USA).

The hydraulic conductivity (L) was calculated, as described [25], using the equation: L = T/[(Ys − Yl) FW], where T is transpiration, FW is the fresh weight of roots and Ys and Yl are the water potential of soil and leaves, correspondingly.

### 2.5. Visualization of Lignin and Suberin

To visualize lignin and suberin with berberine hemisulfate [26], cross-sections were made by vibratome Leica VT1200S (“Leica”, Deer Park, IL, USA) from the segments of the root hair zone (3–4 cm from the root tip) and basal part of the roots on the 6th day after the start of experiments. Sections were stained with berberine hemisulfate (0.1% *w*/*v*) for 1 h, then stained with toluidine blue (0.05% *w*/*v*) for 15 min to enhance intensity of fluorescence and rinsed 2 times with distilled water before embedding in 50% glycerol mixture and covered with a cover slip. Fluorescence of berberine was excited with a 488 nm solid-state laser using a laser scanning confocal microscope Olympus FluoView FV3000 (Olympus, Tokyo, Japan). Fluorescence emission was detected at 520 nm.

### 2.6. RNA Extraction and Quantitative Real-Time Polymerase Chain Reaction (qPCR)

Six days after the start of bacterial treatment, total RNA was extracted from roots using TRIzol™ Reagent (Sigma, Neustadt, Germany) according to the manufacturer’s instructions. DNA was destroyed with DNaseI (Syntol, Moscow, Russia), and the first-strand cDNA was synthesized with M−MLV reverse transcriptase (Fermentas, Waltham, MA, USA) and oligo(dT)15 used as a primer. Further, 2 μL of diluted cDNA was used for qPCR. The primers were designed with PrimerQuest™ Tool based on the cDNA sequences (Table 1).

Quantitative PCR was performed with a set of predefined reagents EvaGreenI (Synthol, Russia) and QuantStudio™ 5 Real-Time PCR System by Thermo Fisher Scientific (Singapore). Ten-fold cDNA dilution series were used to confirm the efficiency of each pair of primers and reliability of the fold changes. Hv α-tubulin (GenBank Accession No. AK250165) was used as an internal reference for the real-time qPCR analysis. Gene expression was quantified with QuantStudio™ 5 Real-Time PCR System by Thermo Fisher Scientific (Singapore). Each experimental treatment was assayed in three biological replicates. 

### 2.7. Generation of Antibodies against AQPs

Polyclonal rabbit antibodies for *HvPIP2s* were obtained with synthetic oligopeptides corresponding to the sequences of amino acids in the N-region of *HvPIP2;1* [17], *HvPIP2;2* [18], *HvPIP2;3/4* [19] and *HvPIP2;5* [20].

### 2.8. Immunolocalization of Aquaporins, IAA and ABA

Immunolocalization was carried out as described [20] on root sections prepared from basal part of roots on 6th day after bacterial treatment. Short root segments were fixed in a solution of 1-ethyl-3-(3-dimethylaminopropyl) carbodiimide (Merck, Darmstadt, Germany) and then with paraformaldehyde (Riedel de Haen, Seelze, Germany) and glutaraldehyde (Sigma, Neustadt, Germany) for a night. Specific rabbit antisera raised against ABA [20], IAA [19] and HvPIP2 AQPs were used to immunolocalize these antigens. Root segments were dehydrated in solutions with increasing concentrations of ethanol and then embedded in methacrylate resin (Electron Microscopy Sciences, Hatfield, PA, USA). Sections, obtained with a rotation microtome (HM 325, MICROM Laborgeräte, Walldorf, Germany), were placed on slides and treated for 30 min with PBS (phosphate-buffered saline) (1×, pH 7.4) containing 0.2% gelatin and 0.05% Tween 20 (PGT), washed with distilled water and incubated with polyclonal rabbit anti-HvPIP2 sera (1:100 dilution) or anti-ABA (dilution 1:50) sera or anti-IAA (dilution 1:50) sera at 4 °C overnight. Slides were washed in PBS with 0.05% Tween-20 and incubation with secondary antibodies against rabbit IgG conjugated to Alexa Fluor 555 (Invitrogen, Rockford, IL, USA) The slices were washed with PBS, covered with a cover slip and then imaged by confocal microscopy using an Olympus FV3000 Fluoview (FV31-HSD) and laser excitation line of 561 nm. Fluorescence emission was detected at 568 nm in integration frame mode for imaging with a count of 2. The difference in intensity of staining was displayed by color-coded heatmap.

### 2.9. Membrane Lipid Peroxidation

The amount of malondialdehyde (MDA—the product of peroxidation of membrane lipids) was determined on the 6th day after inoculation as described [27].

### 2.10. Statistics

Data expressed as means ± SE were calculated in all treatments using MS Excel. The significances of differences were analyzed with Student’s *t*-test (*p* ≤ 0.05).

## 3. Results

Bacterial hormone production, phosphate-mobilizing activity and the ability to hydrolyze chitin were measured (Table 2). *Pseudomonas mandelii* IB-Ki14 was able to synthesize the auxin indole-acetic acid (IAA) and abscisic acid (ABA) and had phosphate-solubilizing activity and weak chitin-hydrolyzing activities. They could grow in 4% NaCl, but these bacteria were unable to antagonize pathogenic fungi.

**Figure 1:**
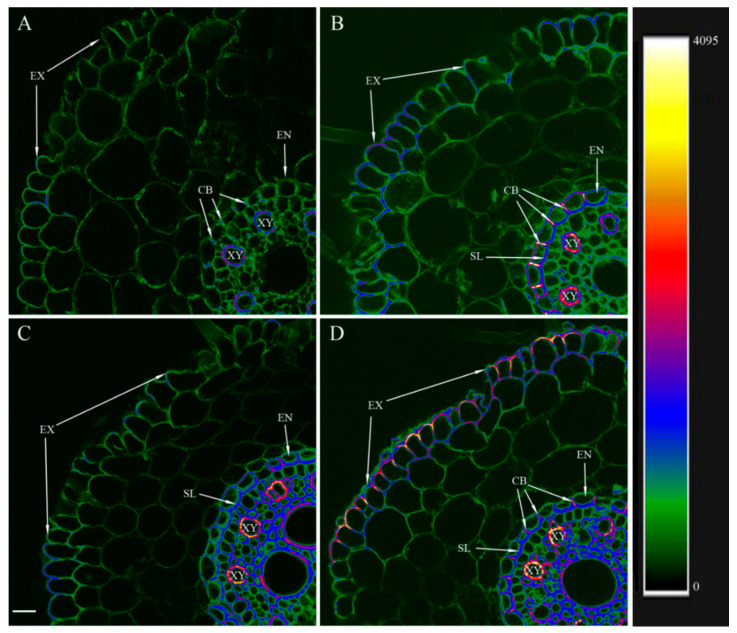
Localization of lignin and suberin and detection of Casparian bands in berberine-stained root cross-sections of the 8-day-old barley plants untreated (**A**,**C**) and treated (**B**,**D**) with *Pseudomonas mandelii* IB-Ki14. Sections were made 6 days after bacterial treatment at 3–4 cm level from the root apex (**A**,**B**) and from basal part (**C**,**D**) of the seminal roots. The scale bar is 25 µm. EX—exodermis, EN—endodermis, SL—suberin lamellae, CB—Casparian bands, XY—xylem.

The results of the berberine staining of transverse sections of barley roots are given in Figure 1, where the intensity of berberine fluorescence recorded at 520 nm is displayed as a color-coded heatmap (from green corresponding to the minimum level of lignin/suberin to yellow/white corresponding to the maximum). The control plants (untreated with bacteria) showed fluorescence intensity mainly as green on sections cut at 3 cm from the root tip (Figure 1A), indicating a low level of cell wall lignification. The fluorescence of the walls of xylem vessels was higher, judging by their red and blue color-coded staining, reflecting the differentiation of xylem vessels with lignin deposited in their walls (Figure 1A). The root sections of the bacteria-treated plants showed a different picture at the same distance from the root tip (3 cm) (Figure 1B). Here, yellow tones appeared in the staining spectrum of the walls of xylem vessels, reflecting an increase in the degree of their lignification. In the endodermis, berberine revealed suberin lamellae and formed Casparian bands. In addition, an increase in fluorescence intensity is also noticeable in the exodermis, which indicates an increase in lignification (Figure 1B). In the basal root region, an increase in berberine fluorescence occurred in both the control and bacteria-treated plants (Figure 1C,D) compared to the root region located closer to the tip. In the control, the fluorescence increased in the area of the endodermis and in the cell walls of the central cylinder, including xylem (Figure 1C). In the bacteria-treated plants, lignification increased not only in these cells but also in the exodermis (Figure 1D).

**Figure 2:**
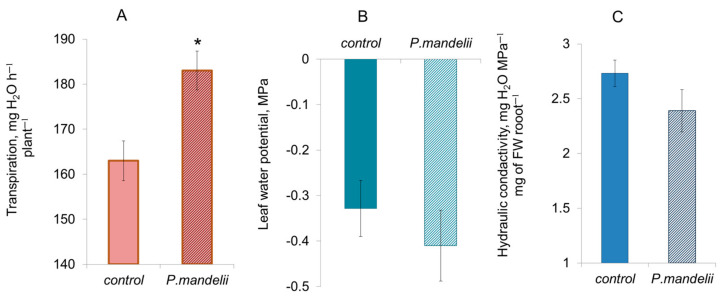
Effects of bacterial treatment with *Pseudomonas mandelii* IB-Ki14 on transpiration (**A**), leaf water potential (**B**) and hydraulic conductivity (**C**) of 8-day-old barley plants 7 days after bacterial treatment (*n* = 6). Means significantly different from the control are labeled with asterisk (*t*-test, *p* ≤ 0.05).

Introducing bacteria into the plant rhizosphere significantly increased their transpiration by approximately 10% (Figure 2A). However, the leaf water potential did not significantly differ between the control and bacteria-treated plants (Figure 2B). Calculating hydraulic conductivity by analogy with Ohm’s law also showed that the plants treated with bacteria had similar values to the control group (Figure 2C).

Bacterial treatment did not significantly influence the level of MDA in plant leaves: it was (24 ± 4) and (23 ± 2) nmol/g.

**Figure 3:**
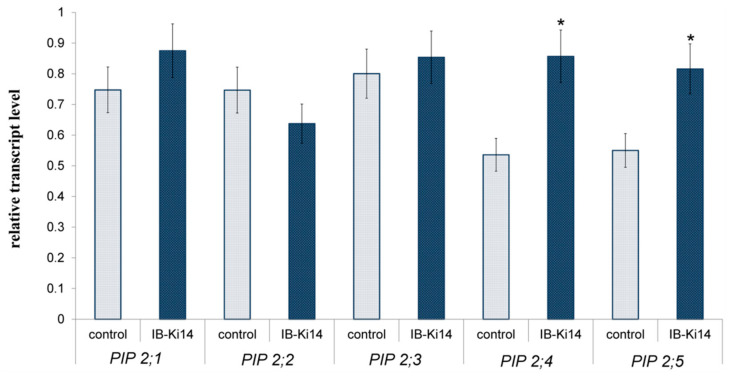
Effect of bacterial treatment with *Pseudomonas mandelii* IB-Ki14 on the content of transcripts of *PIP2* genes in roots of the 8-day-old barley plants untreated (control) and treated (IB-Ki14) with bacterial strain. Roots were sampled 6 days after bacterial treatment. The expression values of aquaporins genes are normalized relative to the barley gene encoding Hv α-tubulin (GenBank Accession No. AK250165). Means (*n* = 3) for transcript level of each gene in bacteria-treated plants significantly different from the control are labeled with asterisk (*t*-test, *p* ≤ 0.05).

The RT-PCR revealed no significant effect of bacteria on the transcript abundance of the genes encoding *HvPIP2;1*, *HvPIP2;2* and *HvPIP2;3* aquaporins, even though *HvPIP2;1* and *HvPIP2;3* tended to increase and *HvPIP2;2* tended to decrease. However, the bacteria-treated plants showed increased levels of *HvPIP2;4* and *HvPIP2;5* transcripts compared to the uninoculated control (Figure 3).

The immunolocalization of *HvPIP2;1* and *HvPIP2;2* with the corresponding antibodies revealed no effect of bacteria on their abundance (Supplementary Figures S1 and S2). *HvPIP2;3* antibodies were able to recognize *HvPIP2;4* [28] due to close similarity between the N-region of *HvPIP2;3* and *HvPIP2;4* proteins, while the *HvPIP2;5* antibodies were more specific and, thus, chosen for further immunohistochemical experiments.

**Figure 4:**
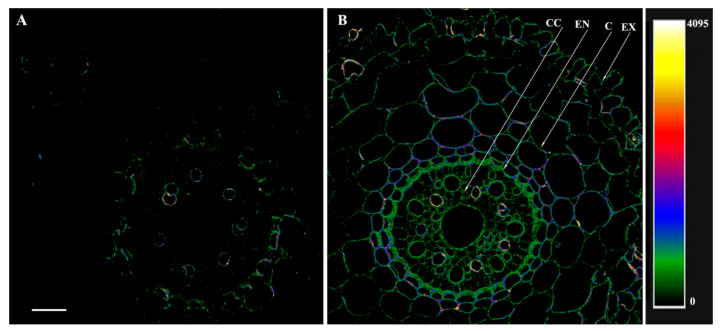
Immunolocalization of PIP2;5 aquaporins on cross-sections from root basal part of 8-day-old barley plants untreated (**A**) and treated with *Pseudomonas mandelii* IB-Ki14 (**B**). Sections were made 6 days after the bacterial treatment. Heatmap shows color-coded fluorescence signal intensities. The scale bar is 50 µm. CC—central cylinder, EN—endodermis, C—cortex, EX—exodermis.

The treatment of the root sections with the serum against HvPIP2;5 and its display with secondary antibodies labeled with Alexa 555 revealed low levels of these aquaporins in the roots of the control plants (Figure 4). In fact, most of the cortical cells could not be visualized as fluorescence was absent in this zone, while fragments of cell contours fluoresced only in the central cylinder and endodermis (Figure 4A). On the contrary, in the plants treated with bacteria, antibodies revealed the presence of HvPIP2;5 aquaporins on the cell periphery of all the root tissues, including the exodermis, with a particularly intense fluorescence being detected around the central cylinder (Figure 4B).

**Figure 5:**
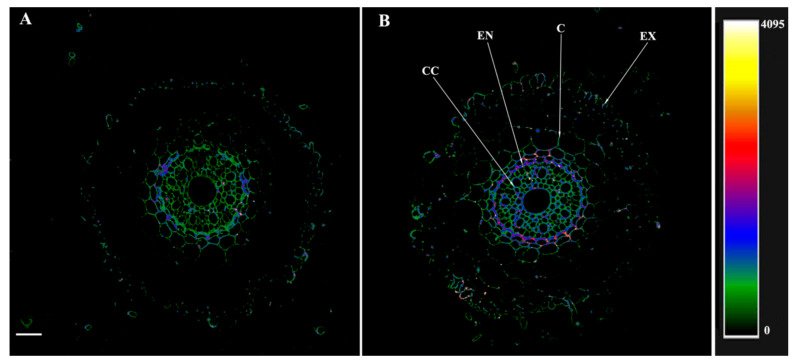
Immunolocalization of IAA on cross-sections from root basal part of 8-day-old barley plants untreated (**A**) and treated with *Pseudomonas mandelii* IB-Ki14 (**B**). Sections were made 6 days after the bacterial treatment. Heatmap shows color-coded fluorescence signal intensities. The scale bar is 50 µm. CC—central cylinder, EN—endodermis, C—cortex, EX—exodermis.

Treating the root sections with antibodies to IAA revealed this hormone was mainly present in the central cylinder and inner cortex layer of the control plants. Other cells of the cortex showed no fluorescence. Blue-coded staining indicated an increased content of this hormone in distinct endodermal cells but only weak emission in the exodermis, indicating less IAA in the cells of this zone (Figure 5A). In the bacteria-treated plants, fluorescence appeared in the cortex and increased in the endodermis, central cylinder and exodermis, reflecting increased auxin concentrations (Figure 5B). In the endodermis zone, the appearance of a red color (in the bacteria-treated plants) instead of the blue color (in the control plants) indicated increased auxin levels.

**Figure 6:**
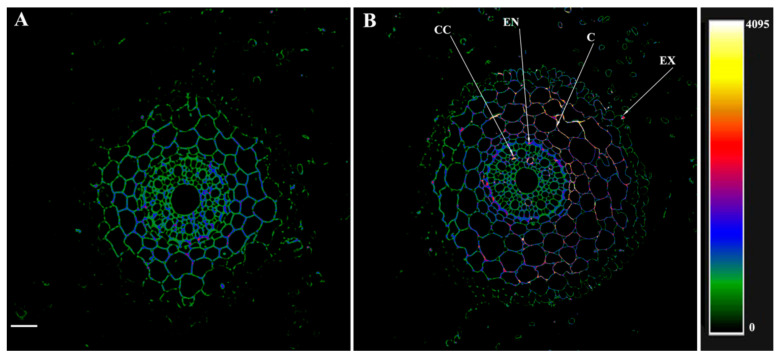
Immunolocalization of ABA on cross-sections from root basal part of 8-day-old barley plants untreated (**A**) and treated with *Pseudomonas mandelii* IB-Ki14 (**B**). Sections were made 6 days after the bacterial treatment. Heatmap shows color-coded fluorescence signal intensities. The scale bar is 50 µm. CC—central cylinder, EN—endodermis, C—cortex, EX—exodermis.

ABA immunolocalization with specific antibodies revealed this hormone was present both in the central cylinder and in the cortex of the control plant roots (Figure 6A). Only the exodermis cells remained poorly distinguishable due to the low level of emission (and, accordingly, the low level of ABA). With bacterial treatment, the fluorescence increased in all the areas of the cortex and appeared in the exodermis, indicating increased levels of this hormone (Figure 6B).

## 4. Discussion

The promotion of plant growth by some rhizosphere bacteria is believed to be due to their ability to synthesize plant hormones, increase the availability of nutrients for plants (e.g., by increasing solubility of phosphates) and to defend plants from pathogens [29]. *Pseudomonas mandelii* IB-Ki14 had no antagonistic activity on fungal pathogens and, although bacterial phosphate-mobilizing activity was detected, it is unlikely to be important in the present experiments, where plants were grown in sand culture with nutrient solution applied. The effects of the studied bacteria on plant growth and development can be mainly explained by their ability to produce hormones.

Staining root sections with berberine revealed that bacteria increased the lignification of cell walls, which was most pronounced in the endodermis and exodermis. The appearance of suberin lamellae in the endodermis closer to the root tip of the bacteria-treated plants indicates accelerated formation of Casparian bands. These results demonstrate that *Pseudomonas mandelii* IB-Ki14 accelerated the formation of, and strengthened, apoplastic barriers. Previously, we detected that this microbe reinforced the apoplastic barriers in salt-stressed wheat plants [5], but the present research also confirms this effect under optimal conditions. Under salinity stress, the formation of apoplastic barriers is accompanied by a decrease in hydraulic conductivity [30,31], which limits the mass flow of toxic ions into the plant with the transpiration stream. Apoplastic barriers also protect plants from pathogen infection [31]. However, in the absence of salinity, the reduced ability of roots to conduct water can adversely affect bacteria-treated plants. Nevertheless, the leaf water potential did not decrease in the bacteria-treated plants despite their increased transpiration rate. An absence of MDA accumulation confirmed that they did not experience oxidative stress [32]. Calculations of hydraulic conductivity showed no decrease in plants treated with bacteria. Since aquaporins significantly contribute to the overall root water transport, it was important to check whether bacterial treatment affected the level of aquaporins and the expression of the genes encoding them. 

The PCR results did not reveal any changes in the abundance of *HvPIP2;1* and *HvPIP2;2*, consistent with the results of immunolocalization of these aquaporins with the corresponding antibodies (Supplementary Figures S1 and S2). Antibodies against the N-region of HvPIP2;3 were not sufficiently specific and showed cross-reactivity with HvPIP2;4, apparently due to the similarity in their primary sequence [28]. Therefore, this work focused on PIP2;5 aquaporins, with bacteria increasing the level of these aquaporins, especially in the region of the endodermis and exodermis, where the formation of apoplastic barriers increased the contribution of aquaporins to overall root water transport. This increased abundance of aquaporins could compensate for the decline in the hydraulic conductivity of the apoplastic pathway brought about by the reinforcement of apoplast barriers as a result of bacterial treatment.

It was important to find out how bacterial treatment could affect the formation of apoplastic barriers and abundance of aquaporins. Previously, ABA stimulated the formation of apoplastic barriers in pea plants [33] and increased aquaporins activity in many species [8,20,34]. In the present work, immunolocalization showed an increased abundance of ABA in the root sections of the bacteria-treated plants, which was most noticeable in the region of the endodermis and exodermis. In these regions, bacteria increased the deposition of lignin and suberin but also increased the level of PIP2;5 aquaporins. Taken together, this suggests that ABA is involved both in stimulating the formation of apoplastic barriers and in compensating for a decrease in the conductance of the apoplastic pathway by the increased abundance of aquaporins. 

The ability of bacteria to produce hormones, and especially auxins, is well-known (Ref. [29] and references therein). IAA was also detected in the bacterial culture medium, suggesting that the bacteria-induced increase in root auxin concentrations was due to plant uptake of bacteria-produced hormones. This *Pseudomonas* strain also produced ABA even if the levels in the bacterial culture media were low. Nevertheless, bacteria with the same level of ABA production are considered to augment plant ABA levels [35]. Furthermore, bacteria could increase the root ABA levels by stimulating plant ABA synthesis. Recently, we have shown an increase in the expression level of the *NCED* gene responsible for ABA synthesis in barley plants treated with *Bacillus subtilis* IB22 [36]. Auxins can also up-regulate this rate-limiting gene responsible for ABA synthesis [37]. Thus, auxins produced by bacteria could increase plant ABA levels, thereby indirectly affecting the formation of apoplast barriers and abundance of aquaporins. A direct effect of auxins on the formation of apoplast barriers is also possible since this hormone is involved in suberin metabolism [38]. While there is a lack of data on the possible direct effect of auxins on aquaporins, these hormones are indicated to decrease rather than increase the activity of aquaporins [39].

Our results and analysis of the literature data suggest that the bacterial mediation of auxin accumulation is directly related to the formation of Casparian bands and not with the content of aquaporins. The increased abundance of PIP2;5 AQPs was more likely linked with ABA accumulation (a hormone known to increase AQPs abundance). Bacteria could trigger ABA accumulation in planta by either producing this hormone or up-regulating the plant genes responsible for ABA synthesis in response to bacterial auxins.

## Figures and Tables

**Table 1:** Sequences of primers used for qRT-PCR (annealing temperature 60 °C).

Genes	Strand	5′ to 3′ Primer Sequences	GenBank Accession Number
*HvPIP2;1*	Forward	GGGTCCTACAGGAGCAACTAA	AB009307
	Reverse	GGCCTTCTTCTTCGCACATATC	
*HvPIP2;2*	Forward	GTCAAGGGCATCATGAAGGA	AB377269
	Reverse	TGTAGACGAGGACGAAGGT	
*HvPIP2;3*	Forward	TGACCAAGTGGTCCCTGTA	AB275280
	Reverse	CTGGTGCTTGTACCCAATGA	
*HvPIP2;4*	Forward	GTCATGTACGCATCCAGGTAA	AB219525
	Reverse	GGACGTATGCTTGGGAGTAAG	
*HvPIP2;5*	Forward	CTGTGTCCATCCATCCAAAGA	AB377270
	Reverse	CAGCTGCCGACACATAAATAAC	
Hv α-tubulin	Forward	GAGCGTCTCTCTGTTGACTATG	AK250165
	Reverse	TGGACAGGACACTGTTGTATG	

**Table 2:** Characterization of the strain *Pseudomonas mandelii* IB-Ki14.

Characteristic		
IAA concentration, ng/mL in the culture medium		220
ABA concentration, ng/mL in the culture medium		5
Phosphate-solubilizing activity, index of solubilization		
	Ca_3_(PO_4_)	2.7
	Al_3_PO_4_·3H_2_O	1
	FePO_4_·2H_2_O	only under the colony
	phytin (C_6_H_6_Ca_5_MgO_24_P_6_)	2.8
Chitin hydrolysis		weak
Cellulose hydrolysis		no
Antagonism against *B. sorokiniana*, *F. culmoruim*, *F. oxysporm*, *F. solani*		absence
Growth in presence NaCl, 1–10%		4

## Data Availability

Not applicable.

## Supplementary Materials

The following supporting information can be downloaded at: https://www.mdpi.com/article/10.3390/microorganisms10050935/s1, Figure S1: Immunolocalization of PIP2;1 aquaporins on cross-sections from root basal part of 8-day-old barley plants untreated (A) and treated with Pseudomonas mandelii IB-Ki14 (B). The scale bar is 100 μm. CC—central cylinder, EN—endodermis, C—cortex, EX—exodermis.; Figure S2. Immunolocalization of PIP2;2 aquaporins on cross-sections from root basal part of 8-day-old barley plants untreated (A) and treated with Pseudomonas mandelii IB-Ki14 (B). The scale bar is 100 μm. CC—central cylinder, EN—endodermis, C—cortex, EX—exodermis.
