# Supplementary material for: The Effects of Rhizosphere Inoculation with Pseudomonas mandelii on Formation of Apoplast Barriers, HvPIP2 Aquaporins and Hydraulic Conductance of Barley
Source: Microorganisms. 2022 Apr 29;10(5):935. doi: 10.3390/microorganisms10050935 (PMC9147626; doi:10.3390/microorganisms10050935)
Supplement: Supplementary file 1 [file microorganisms-10-00935-s001.zip › microorganisms-1681646-supplementary.pdf]

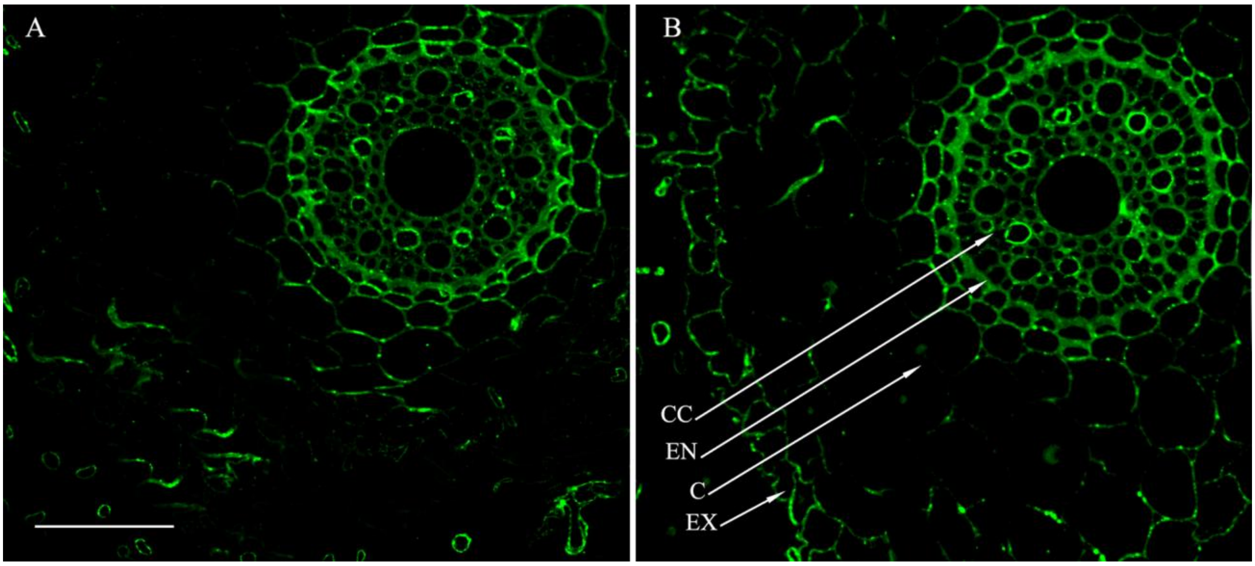

Figure S1. Immunolocalization of PIP2;1 aquaporins on cross-sections from root basal part of 8-day-old barley plants untreated (**A**) and in treated with *Pseudomonas mandelii* IB-Ki14 (**B**). The scale bar is 100 µm. CC – central cylinder, EN – endodermis, C – cortex, EX – exodermis.

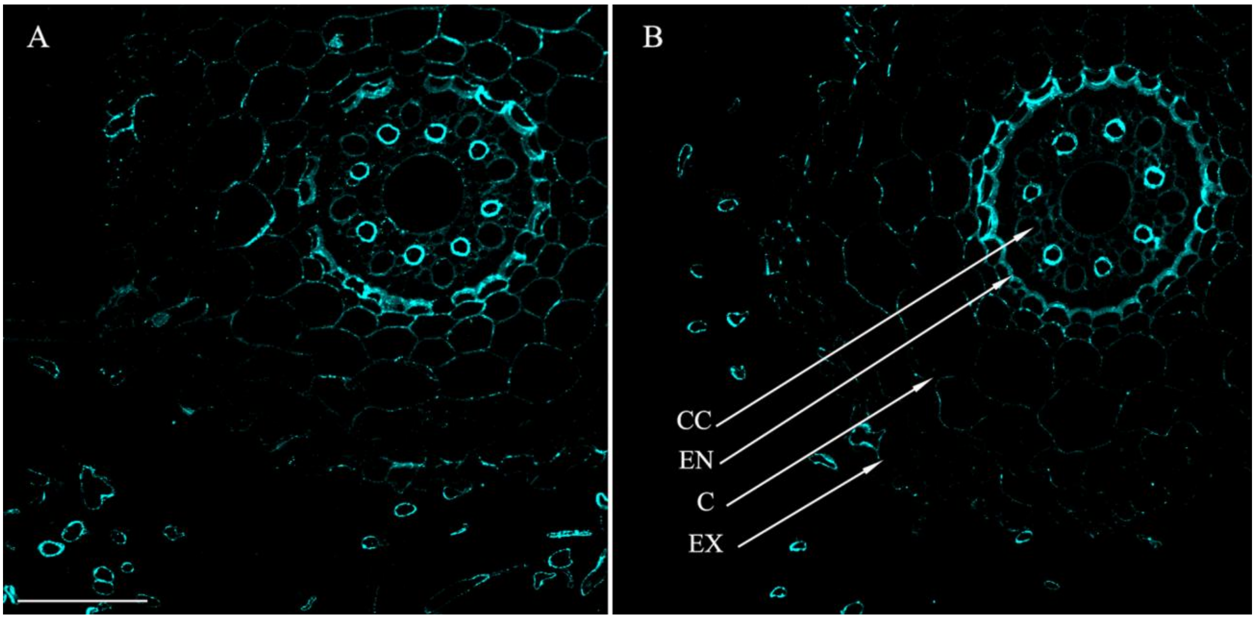

Figure S2. Immunolocalization of PIP2;2 aquaporins on cross-sections from root basal part of 8-day-old barley plants untreated (**A**) and in treated with *Pseudomonas mandelii* IB-Ki14 (**B**). The scale bar is 100 µm. CC – central cylinder, EN – endodermis, C – cortex, EX – exodermis.
